# Linking physiological coordination and behavioral pattern complexity to team adaptation and performance in medical simulations

**DOI:** 10.3389/fnrgo.2026.1743312

**Published:** 2026-10-22

**Authors:** Elwira A. Hałgas, Joyce H. D. M. Westerink, Travis J. Wiltshire, Sonja Rispens, Kyana H. J. van Eijndhoven, Annemarie Fransen, Josette M. P. Gevers

**Affiliations:** 1 Industrial Engineering & Innovation Sciences, Eindhoven University of Technology, Eindhoven, Netherlands; 2 Department of Digital Engagement, Behaviour, and Cognition, Philips Research, Eindhoven, Netherlands; 3 Department of Computational Cognitive Science, Tilburg University, Tilburg, Netherlands; 4 Maxima Medisch Centrum, Veldhoven, Netherlands; 5 Zuyderland Medisch Centrum, Sittard-Geleen, Netherlands

**Keywords:** behavioral interaction patterns, physiological coordination, simulation-based training, team adaptation, team performance

## Abstract

**Introduction:**

In dynamic, high-stakes environments, such as medical emergencies, effective teamwork and team performance rely on team adaptation. Team Behavioral Interaction Patterns (i.e., recurring sequences of team behaviors; TBIPs) have been proposed as markers of adaptive functioning, while physiological coordination among team members may offer an unobtrusive, real-time proxy for such behavioral dynamics. The current paper presents an investigation of whether TBIPs are (1) related to self-reported team adaptation and rated performance, and (2) reflected in physiological coordination. The paper advances the potential use of wearable technology for (near-) real-time team monitoring.

**Methods:**

We conducted a quasi-experimental study with 19 medical teams (*N* = 150) during simulation-based medical emergency team training. Teams were video-recorded and wore wrist-worn sensors capturing electrodermal activity (EDA) and heart rate (HR). Based on video data, we extracted TBIPs via T-pattern detection. We assessed the complexity of TBIPs and calculated physiological coordination (synchrony and regularity) for each pattern. Team adaptation was measured via self-report, and team performance was rated by trainers. We related physiological coordination to team adaptation and team performance, while accounting for the complexity of TBIPs.

**Results:**

Regression analyses showed no association between TBIP complexity and team performance. Multilevel models revealed that the relationship between physiological synchrony (HR and EDA) and team adaptation, and that between EDA regularity and team performance, depended on TBIP complexity. Specifically, better team adaptation was related to higher HR synchrony during low-complexity TBIPs and lower EDA synchrony during high-complexity TBIPs, whereas better team performance was linked to higher EDA regularity during high-complexity TBIPs.

**Conclusion:**

Our findings suggest that behavioral pattern complexity moderates the relationship between physiological coordination and team outcomes. This interplay between behavioral and physiological data highlights the value of combining both modalities to understand and potentially support adaptive team functioning.

## 1 Introduction

A multitude of critical functions in society depend on the viability of action teams, such as medical teams and police enforcement teams. These teams are specialized units operating in time-constrained and high-stakes environments requiring improvisation and cooperation (Bell et al., 2018; Waller and Uitdewilligen, 2013). Their performance depends on the ability of team members to engage in collective, interdependent actions and mutual adaptation (DeChurch and Mesmer-Magnus, 2010b; Katz-Navon and Erez, 2005).

*Team adaptation* is the dynamic process in which a team recognizes the need for change, adjusts relevant team processes accordingly, and recovers from changes in the task or environment (Baard et al., 2014; Burke et al., 2006; Georganta et al., 2021a; LePine, 2005; Rico et al., 2021). Team adaptation requires teams to assess situational demands, modify interaction patterns, and coordinate responses accordingly (Georganta et al., 2021b; Gorman et al., 2010b).

Coordination can be mapped onto two dimensions: *content* (action vs. information) and *mode of interaction* (explicit vs. implicit; Kolbe et al., 2013), effectively creating a four-quadrant space into which any single behavior can be located. Each quadrant captures a distinct behavioral mode: explicit action coordination, explicit information coordination, implicit action coordination, and implicit information coordination. Action coordination refers to performing independent activities within an appropriate time frame and in the correct order, while information coordination involves the exchange of information among team members and discussing the implications of action for the task at hand. Coordination is considered explicit when it is used intentionally and straightforwardly, typically verbally or through written communication, and is usually easy to understand (Espinosa et al., 2004; Kolbe et al., 2017; Kolbe and Boos, 2019). In contrast, implicit coordination relies on awareness and anticipation of the actions and needs of other team members and concurrent modification of one's own actions (Kolbe et al., 2017; MacMillan et al., 2004; Rico et al., 2008; Toups Dugas and Kerne, 2007). For successful implicit coordination, team members must have a common understanding of the task situation and agree on the actions to be taken, i.e., they must have so-called shared situation awareness and shared mental models (DeChurch and Mesmer-Magnus, 2010b,a).

Teams shift between explicit and implicit coordination, and between action- and information-related coordination, depending on what is required for performance, considering limited resources and time (Grote et al., 2018; Rico et al., 2019). Such changes in coordination behavior are viewed as manifestations of team adaptation, signaling the team's awareness and acknowledgment of the need to adjust its strategy in response to a novel task situation (Kolbe et al., 2011). The changes in coordination modes or behavior may take the form of so-called Team Behavioral Interaction Patterns (TBIPs; e.g., David et al., 2024).

Team Behavioral Interaction Patterns (TBIPs) are emergent, structured, and repetitive cycles of verbal and non-verbal actions that develop through repeated team interactions (Feldman, 2000; Zellmer-Bruhn et al., 2004). Teams often rely on TBIPs to navigate situational demands requiring team adaptation. These patterns are often reinforced through training and repeated practice, enabling teams to internalize shared expectations and behaviors (Feldman, 2000). For example, a medical team may establish TBIPs through training to develop a routine for managing life-threatening emergencies, such as anaphylactic shock. Implicit coordination patterns emerge from the shared understanding of task demands and roles, requiring no explicit verbal communication, such as synchronized actions during a procedure (Rico et al., 2008). As such, TBIPs can increase a team's efficiency by increasing team members' predictability and establishing routines that facilitate team coordination (Zellmer-Bruhn et al., 2004). Yet, overreliance on TBIPs may hinder team adaptation when preexisting patterns should be updated or new ones should be created to align with changing situational requirements (Gorman et al., 2010b). In such cases, teams must go beyond learned routines and develop new interaction patterns that better fit changing demands (Hoogeboom and Wilderom, 2020; Rico et al., 2021). The ability to adjust interaction patterns in response to disruptions is a hallmark of high-performing teams (Grote et al., 2018).

One defining quality of TBIPs is their complexity, reflecting the structural elaborateness with which behavioral actions are combined and organized over time. In T-pattern analysis, simpler behavioral sequences recursively combine into increasingly complex higher-order structures (Magnusson, 2000). More complex interaction patterns may therefore comprise more behavioral elements organized in more deeply nested hierarchical structures (Hoogeboom and Wilderom, 2020). In the present study, we operationalize this structural complexity using the number of hierarchical levels within a TBIP. For example, a simple sequence of two behaviors (e.g., question-answer) would constitute a low-complexity TBIP (complexity equal to one), whereas a pattern in which such lower-level sequences are nested within a broader interaction structure {e.g., [(question-answer)-clarification-(question-answer)-decision] would reflect a higher level of TBIP complexity (complexity equal to two}. As such, TBIP complexity captures the structural richness of temporal interaction patterns, whereby simpler TBIPs may support rapid responses, while highly complex TBIPs may risk slowing down decision-making (Stachowski et al., 2009).

TBIP complexity can also be seen as a context-dependent feature of continuously adjusting team processes. Consistent with the Law of Requisite Variety, adaptive systems require sufficient internal variety to match the complexity imposed by the environment (Ashby, 1957). That is, the system (i.e., the team) may produce sufficient internal variety to match the variety imposed by the environment, for example, by increased reorganization at the team level due to greater environmental demands (Grimm et al., 2023). Related work on complexity matching similarly suggests that interacting systems dynamically adapt their complexity during coordination processes (Mahmoodi et al., 2019). In the context of TBIPs, teams may therefore match the complexity of patterns to the demands imposed by the situation. For example, when task demands are familiar, teams may rely on less complex, well-practiced patterns, while more novel or uncertain situations may require greater behavioral variety and more complex TBIPs. In fact, empirical evidence aligns with this perspective, as high-performing teams showed shorter and less complex TBIPs in simulated nuclear plant emergencies requiring teams to follow a structured protocol (Stachowski et al., 2009), whereas longer and more complex TBIPs were identified in the responses to the crisis during the Apollo 13 mission (David et al., 2024).

Yet, the understanding of how TBIP complexity relates to team adaptation and performance remains limited, as prior findings have been inconsistent, highlighting the need to further examine the role of TBIP complexity in team adaptation and team performance, leading to our first research question:

*RQ1. Does the complexity of team behavioral interaction patterns relate to team adaptation and team performance?*

While existing training for adaptive responses to crisis situations is extremely valuable, action teams could benefit even further from receiving (near) real-time feedback on their adaptive functioning as it unfolds. Such feedback is not yet feasible, but recent technological advances point toward possible future applications. Building on evidence from psychophysiology that signals such as heart rate and skin conductance reflect the emotional and cognitive states of individuals (Cacioppo et al., 2016), a promising approach involves combining measures of team members' physiological signals to reflect the state of a team as a whole.

From a Complex Systems Theory perspective, teams can be understood as complex dynamic systems in which interactions among members unfold and can be measured across multiple levels of the system (e.g., behavioral, cognitive, physiological; Abraham and Shaw, 2008; Arrow et al., 2000; Gorman, 2014; Wiltshire et al., 2019). Within such systems, individual team members and the sub-systems within them continuously influence one another, giving rise to emergent team-level phenomena that cannot be understood solely from individual components (Arrow et al., 2000; Gorman et al., 2016). A key premise of this perspective is that processes occurring at one level of the system (e.g., physiology) may provide insight into processes unfolding at other levels (e.g., behavior; Amazeen, 2018; van Eijndhoven et al., 2024a,b). Building on these principles, the Team Coordination Dynamics Framework applies a complex systems perspective to team functioning by proposing that coordination emerges through dynamic coupling processes across multiple system levels (Gorman et al., 2010a). Within this framework, the coupling of physiological signals across team members, referred to as team physiological coordination, may function as a marker of unfolding team processes and states that emerge during collaboration.

Team physiological coordination can be derived from multiple signals, such as heart rate and skin conductance (Cooke et al., 2013; Gorman et al., 2010a) and calculated using various methodologies, such as synchrony or regularity (Amon et al., 2019; Gorman and Wiltshire, 2024; Guastello et al., 2022; Hałgas et al., 2022; Hudson et al., 2023). *Synchrony* refers to a state in which the physiological signals of team members align and change simultaneously, such as a co-occurring increase in heart rate (Palumbo et al., 2017). *Regularity* refers to how team members' physiological responses occur repeatedly and predictably over time, reflecting the extent to which the team revisits the same sequences of states (Wallot and Leonardi, 2018).

Prior research demonstrates that measures of team physiological coordination are linked to various dimensions of team functioning, including cognitive, affective, and behavioral processes involved in team adaptation (Cooke et al., 2013; Haataja et al., 2018; Hałgas et al., 2022). Importantly, physiological coordination may capture emergent interactional processes that cannot be inferred from individual physiological states alone. Indeed, physiological coordination has been shown to provide additional explanatory and predictive value for team information processing, collective decision-making, and team performance beyond individual physiological measures (Li et al., 2025; Sharika et al., 2024). Building on the premises of Complex Systems Theory and the Team Coordination Dynamics Framework, these findings suggest that synchrony and regularity in heart rate and skin conductance may serve as nonverbal markers of emergent team-level processes (Gorman et al., 2010a; Tognoli et al., 2020; Wiltshire et al., 2019). In particular, when these forms of physiological coordination occur during TBIPs, they may reflect ongoing adjustments in cognitive, affective, and behavioral processes as teams collectively adapt to unfolding demands. As such, physiological coordination may not only capture important team adaptation dynamics, but may also be linked to team performance during critical events. At the same time, the relationship between behavioral and physiological coordination is likely reciprocal. Behavioral interaction dynamics, such as speaking patterns and turn-taking, may shape physiological coordination through shared breathing, posture, and articulation processes (Shockley et al., 2007), while physiological coordination may simultaneously reflect the degree to which team members are aligned in their responses to unfolding task demands.

Thus, understanding how physiological coordination during various types of TBIPs relates to team adaptation and team performance can offer valuable insights for the development of team monitoring and feedback systems. Specifically, because TBIP complexity may be linked to team adaptation and performance, it may also shape how physiological coordination relates to team adaptation and performance. For this reason, the current paper presents an investigation of the relationship between team adaptation, TBIPs derived from observational data, and measures of team physiological coordination calculated from skin conductance and heart rate, leading to our second research question:

*RQ2. Does physiological coordination during TBIPs relate to team adaptation or team performance, and does this relationship depend on the complexity of the TBIP?*

To empirically examine these research questions, we conducted a quasi-experimental study with medical teams engaging in simulation-based team training for high-stakes medical emergency scenarios.

## 2 Methods

### 2.1 Study design

We conducted a quasi-experimental study in hospitals A and B in the Netherlands. Participants were medical professionals taking part in a simulation-based team training consisting of several training sessions, each including a simulation and a team debrief at the end. The simulation training was part of their continuous professional education. A medical scenario was presented at each session, and a medical case was simulated in which trainees attended to either mannequins or actors serving as their patients. We recorded audio and video of the training; we also took measures of participants' heart rate, skin conductance, arm movement, and temperature with wrist-worn wearable devices. Moreover, trainees completed questionnaires assessing their team adaptation, while trainers assessed team performance before the debriefs.

### 2.2 Participants

A total of 20 teams from two hospitals (A and B) participated in the study, completing 43 scenarios. The study involved 150 medical professionals as trainees (141 females, 9 males; age range: 18–64) and two medical professionals (one female, one male) as trainers. All participants were practicing medical professionals recruited from relevant hospital departments and provided informed consent prior to participation. Participants' professional experience ranged from 0 to 45 years (*M* = 10.76, *SD* = 10.62). Trainees were assigned by the trainer to multidisciplinary teams consisting of six to ten members (*M* = 8.25, *SD* = 1.27). Teams were composed exclusively of healthcare professionals. Across all teams, trainees included three midwives, 80 nurses (emergency care, pediatrics, gynecology, obstetrics), four physician assistants (pediatrics), 14 residents (pediatrics, gynecology), and 47 specialists (emergency care, pediatrics, gynecology, obstetrics). Average team experience ranged from 5.39 to 18.30 years (*M* = 10.95, *SD* = 3.48). Due to data quality issues, including video availability and physiological data completeness, data from 19 teams and 37 scenarios were included in the analysis.

In hospital A, teams generally consisted of an obstetrics and gynecology specialist, an obstetrics and gynecology resident, a (clinical) midwife, and nurses. Not all team members participated in both scenarios. Rather, participation varied across simulations, such that only a subset of the full team took part in a given scenario, with participant composition differing across scenarios. In hospital B, team composition remained relatively stable across scenarios, with team members generally participating together across simulations. Teams consisted of a pediatric specialist, pediatric residents, pediatric nurses, and pediatric emergency care, including emergency nurses. During some sessions, members from other teams were present as observers and viewed the training from a separate area of the room; however, these individuals did not actively participate in the simulation. In both hospitals, at least one medical specialist and one nurse were always involved.

### 2.3 Procedure

Before the training day, the trainer sent participants an email with study information and an information letter. They were also asked to read and sign the consent form if they agreed to participate in the study. On the scheduled training day, participants were welcomed by the trainer, introduced to the research staff, and asked whether they had questions regarding the study. Sessions took place in the hospital rooms where all necessary medical equipment was available. Each hospital had its own training scheme, and scenarios differed per team; however, all scenarios included a medical emergency that trainees needed to address.

In each hospital, after the trainer announced the participants for the first scenario, all wearables were synchronized with each other, and the experimenter added a timestamp to one wearable device to ensure that the audio-video recordings could be aligned later. The participating trainees were then asked to put on wearable devices and to fill in a demographic questionnaire. Then, the experimenter turned on the camera in the room and either left the room or moved to the side, after which the first scenario was started.

In Hospital A, where obstetrics and gynecology teams participated, each team completed two simulation scenarios: breech presentation at birth (*M* duration = 18 m 41 s, range = 14 m 29 s-23 m) and pneumonia with septic shock (pneumosepsis) (*M* duration = 15m 22 s, range = 11 m 39 s-17 m 32 s). The same two scenarios were used across teams, and both required interdisciplinary communication, coordination, decision-making under time pressure, and escalation of care. The scenario started with the first-line healthcare provider (e.g., midwife) attending to the patient. Following an initial assessment, care was escalated to involve additional clinicians, generally in the following order: resident or clinical midwife, nurses, and medical specialist. After the diagnosis was correctly identified and the emergency was managed by the team, the scenario was considered complete.

In Hospital B, where pediatrics and emergency room teams participated, each team completed three scenarios. Scenarios involved a range of pediatric emergency situations, including supraventricular tachycardia (SVT) (*M* duration = 20 m 52 s, range = 15 m 12 s-25 m 53 s), hypovolemic shock (*M* duration = 17 m 27 s, range = 14 m 55 s-19 m 51 s), and bronchiolitis with respiratory failure (*M* duration = 14 m 53 s, range = 11 m 29 s-17 m 39 s). Similar to hospital A, the same scenarios were used across teams and teams managed the emergencies differently while still being required to coordinate and communicate. Scenarios generally began with emergency department nurses assessing and managing the patient. As the patient's condition evolved, care was escalated to involve additional clinicians, including pediatric nurses, physician assistants, residents, and pediatric and emergency response specialists. Scenarios concluded once the emergency had been appropriately managed, the patient had stabilized, or transfer to a higher level of care had been arranged.

Upon completion of the first scenario, the trainees and the trainer were asked to fill in questionnaires, and then the trainer debriefed the trainees about the first scenario. After coordinating with the trainer regarding who would participate in the second scenario, the experimenters instructed trainees who would be absent from that scenario to remove their devices for cleaning and redistribution to trainees who would be taking part in their place. Trainees who would participate in both scenarios kept their wearable devices on. Upon completion of the scenario, trainees and trainers were again asked to complete questionnaires before the scenario was debriefed. After all scenarios were completed, the trainer debriefed the trainees about the research and thanked them for their participation.

### 2.4 Measures

#### 2.4.1 Team behavioral interaction pattern complexity

We used the GoPro Max 360 camera to record audio-visual data during the training. Based on these data, team coordination was coded using the Framework for Observing Coordination Behavior in Acute Care Teams (Co-ACT; Kolbe et al., 2013). The framework considers two dimensions, one content-related (action vs. information) and one mode-related (implicit vs. explicit), jointly creating a four-quadrant space. Back-up behavior, or providing unsolicited assistance, is an example of implicit action coordination, whereas giving directions for an action is an example of explicit action coordination. Similarly, talking to the room is considered implicit information coordination, whereas closed-loop communication is an example of explicit information coordination. Prior to coding, the researcher, the trainer from one of the hospitals, and two independent coders reviewed and agreed on the coding scheme. Next, the coders coded the videos for the presence of coordination at 1-s intervals (1 Hz), recording each instance along with its content and mode, assigning it to one of four quadrants and a corresponding subcategory (12 categories in total; Kolbe et al., 2013). In cases where teams temporarily split into sub-teams, coordination behaviors were coded in parallel streams. For T-pattern detection, these were subsequently transformed into a single sequence of first-occurrence events, resulting in a non-overlapping event structure. Moreover, where no coordination was observed, its absence was marked. Throughout the coding processes, coders discussed differences in their annotations and undecided cases. Whenever coding between two coders was not unanimous, the researcher who created the codebook made the final judgment. To calculate intercoder reliability, we calculated Cohen's Kappa (κ) for annotations of 12 scenarios (25% of the sample; O'Connor and Joffe, 2020), resulting in Mean κ = 0.80 for all coordination quadrants. The finalized coding resulted in 1 Hz time series of the team coordination behaviors for each scenario.

To obtain TBIPs, the coded data were then transformed into a format compatible with the THEME6 Edu program, following the guidelines by Magnusson (2000). In the sequence of events, we coded only the first occurrence of each event, as illustrated in Figure 1.

**Figure 1:**
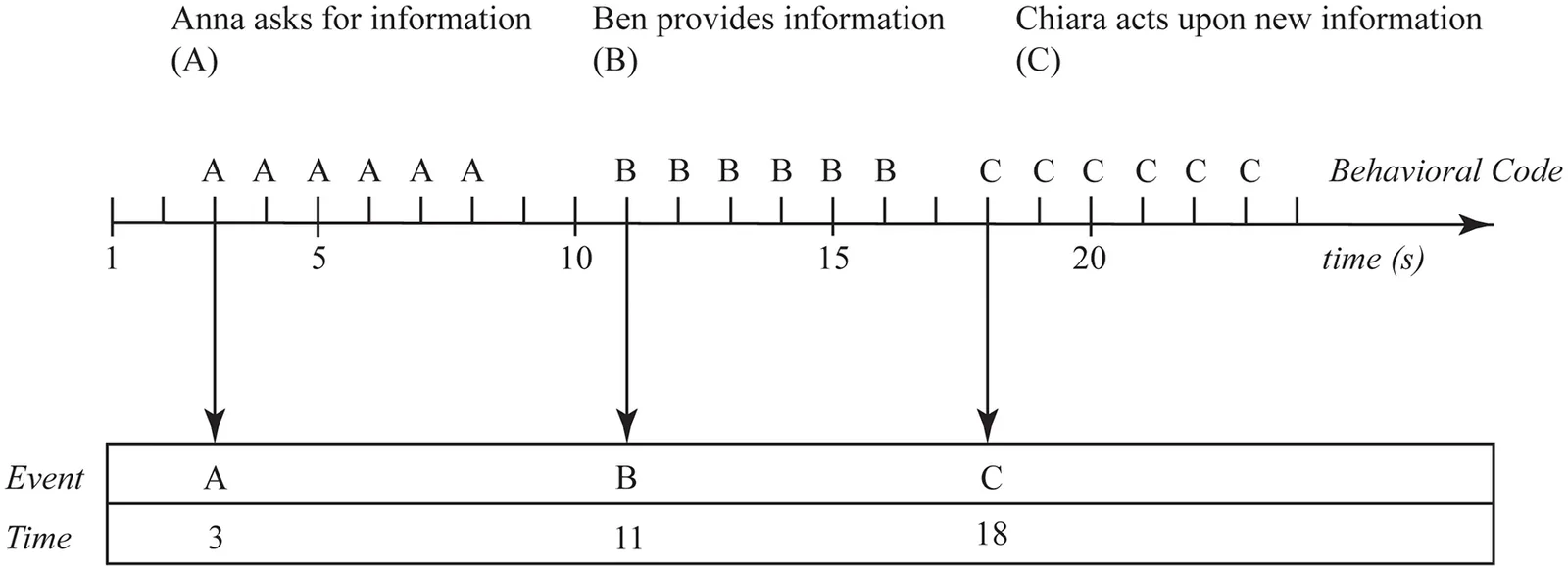
An example of the data extraction process from coded data.

We then used the THEME6 program to identify temporal patterns (T-patterns) within the data (Magnusson, 2000). T-patterns are recurring sequences of events that follow a specific temporal structure, where both the order of events and the intervals between them occur in a non-random, organized way. These patterns have a hierarchical structure (or level), that is, they consist of nested behavioral elements, where higher-level patterns are composed of multiple lower-level behaviors that occur in a specific temporal sequence. For example, a simple pattern may involve a clinician requesting information followed by another team member providing an answer, representing a low-complexity interaction sequence. Higher-complexity patterns emerge when such lower-level sequences become embedded within broader interactions. For instance, a repeated question–answer sequence may be followed by clarification and subsequent decision-making behaviors, creating a nested structure in which smaller recurring behavioral units form components of larger interaction patterns. Figure 2 illustrates this hierarchical organization.

**Figure 2:**
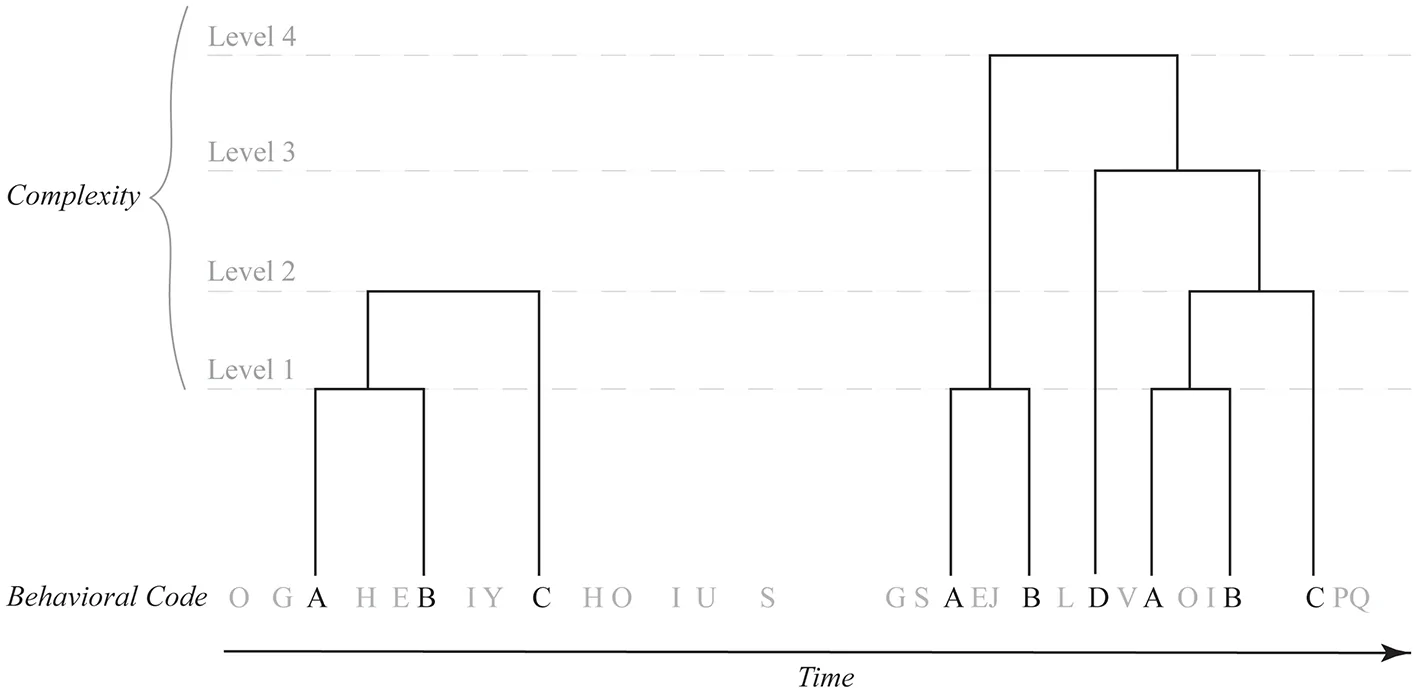
Examples of low- and high-complexity T-patterns illustrating their hierarchical organization. The left pattern represents a pattern of length three and complexity level two. An example of such an interaction would be a clinician explicitly raising a concern about a patient's condition **(A)**, another team member providing relevant information **(B)**, followed by a task-related response from another clinician **(C)**. The pattern on the right represents a higher-complexity pattern of length six and complexity level four. An example of such an interaction would be a clinician raising a concern **(A)**, another team member providing information **(B)**, a third clinician initiating a task-related action **(D)**, followed by an additional cycle of speaking up **(A)**, information exchange **(B)**, and task-related response **(C)**. The higher-complexity pattern contains the lower-complexity sequence as an embedded subpattern, illustrating how behavioral complexity increases through the hierarchical combination of simpler interaction sequences.

Following Rico et al. (2021), we set the minimum number of occurrences for a pattern to three and retained only those patterns that occurred significantly more often than expected under randomized data (*p* < 0.05), as determined by the THEME algorithm (Magnusson, 2000). For each detected pattern, TBIP complexity was operationalized as the number of levels in its hierarchical structure. Since only the beginning of each behavior was coded, the actual duration of a pattern might be slightly longer. As such, we extracted the last instance of the coded behavior for each pattern from the original data and adjusted the actual end time of the pattern accordingly. To address RQ1 and obtain scenario-level metrics, we calculated TBIP complexity as the mean of pattern complexities within each scenario. For RQ2, which involves pattern-level analysis, we used the original, unaggregated complexity values.

#### 2.4.2 Team physiological coordination

We used the Empatica E4 wristband (Empatica Srl, Milan, Italy) placed on the participant's non-dominant wrist to record the photoplethysmogram (PPG) and electrodermal activity (EDA). The PPG signal was recorded at 64 Hz, while skin conductance was sampled at 4 Hz and then resampled for preprocessing at 40 Hz. Both signals were preprocessed with the Neurokit2 tool in Python (Makowski et al., 2021). We applied an additional bandwidth filter (0.7–3.5 Hz) using the Python library HeartPy (van Gent et al., 2019). Heart rate (HR) was then calculated using a sliding window technique based on 120 s of cleaned PPG data and incremented by a step size of 1 s. This resulted in a continuous time series of HR values at 1 Hz and flattened the HR data to some extent. The longer window size for data processing was chosen due to poor data quality. Across all recordings, an average of 3.61% (*SD* = 9.47) of HR windows could not be calculated from the cleaned PPG signal and were replaced using estimates provided by the Empatica E4 software. For electrodermal activity (EDA), we followed the standard Neurokit2 implementation (Makowski et al., 2021). First, the data were upsampled from 4 to 40 Hz to allow the application of a 4th-order Butterworth low-pass filter with a 3 Hz cutoff frequency. Using Neurokit2, the EDA signal was decomposed and the phasic component of EDA (electrodermal responses; EDR), rather than the tonic component (electrodermal level; EDL) was extracted for further analyses. The data were resampled to 4 Hz. The resulting continuous EDR time series was used as input for the analyses.

For each identified TBIP, we calculated the value of team physiological coordination with two methods applied to both heart rate and skin conductance signals. We used coherence to estimate *physiological synchrony* in heart rate and skin conductance (e.g., Reinero et al., 2021) and multidimensional recurrence quantification analysis to estimate *physiological regularity* in heart rate and skin conductance (MdRQA; Wallot et al., 2016). As such, four team physiological coordination metrics (skin conductance synchrony, heart rate synchrony, skin conductance regularity, heart rate regularity) were extracted per TBIP. All physiological signals from team members participating in a given scenario were entered simultaneously into multivariate coordination analyses, yielding indices of collective team-level physiological coordination.

*Coherence* measures how well one signal approximates another by comparing their power spectra through spectral decomposition (White, 1984). It ranges from 0 to 1, with 1 indicating a perfect linear relationship (Winterhalder et al., 2006). We calculated coherence using the multiSyncPy package (Hudson et al., 2023). We further used Multidimensional Recurrence Quantification Analysis (MdRQA), which examines patterns in multivariate time series by identifying recurrent states over time (Wallot et al., 2016). Recurrence is determined based on a similarity threshold, parameter *r*. We used the percentage of *determinism (%DET)*, which quantifies the proportion of recurrence points in a recurrence plot that form diagonal lines, representing predictable sequences of states over time. Higher %DET indicates greater regularity, reflecting more repetition in the data. Following the guidelines by Wallot and Leonardi (2018) we did not embed the data, and the radius (*r*) parameter was calculated for each team such that the recurrence rate was equal to 1%. We used coherence as a measure of *team physiological synchrony* and %DET as a measure of *team physiological regularity*.

To extract the value of physiological coordination for each TBIP, we first followed the methodology registered in our preregistration [see https://osf.io/x9tph]. Physiological coordination, in particular MdRQA, requires an adequate number of observations for reliable detection of recurrence structures (Wallot et al., 2016; Wallot and Leonardi, 2018) as shorter windows may produce unstable estimates. As such, the adequate window length for MdRQA was 60 s. For consistency across methods, we also applied the same window length to the coherence analysis. Based on these considerations, we first excluded patterns shorter than 60 s from the analysis (*N* total = 773; 484 patterns excluded; 289 in sample). However, this deletion resulted in an imbalanced dataset, with the majority of remaining TBIPs (*N* = 235; 81% of the data) sourced from only three teams. Therefore, we decided to only exclude patterns shorter than 30 s (277 patterns excluded), and consequently to also modify our calculation of team physiological coordination per TBIP. Specifically, we decided to include only the first value of physiological coordination for that pattern, meaning that the estimate was based on the first 60 s after the start of each pattern (see Figure 3). Consequently, for TBIPs shorter than 60 s, the physiological coordination window extended beyond the end of the TBIP, thereby including physiological activity occurring during periods in which behaviors outside the identified pattern took place. In addition, for HR, each value within this 60 s window was estimated from the preceding 120 s of PPG data. Thus, our HR-based coordination measures reflect slowly varying changes in HR rather than fine-grained moment-to-moment fluctuations, and also incorporate physiological activity occurring before TBIP onset. Taken together, HR-based coordination estimates could therefore reflect more slowly varying physiological activity occurring shortly before the onset of, and after the end of, the corresponding TBIP. This approach resulted in data being extracted from all teams (*M* = 14.59, *SD* = 20.72 patterns per scenario).

**Figure 3:**
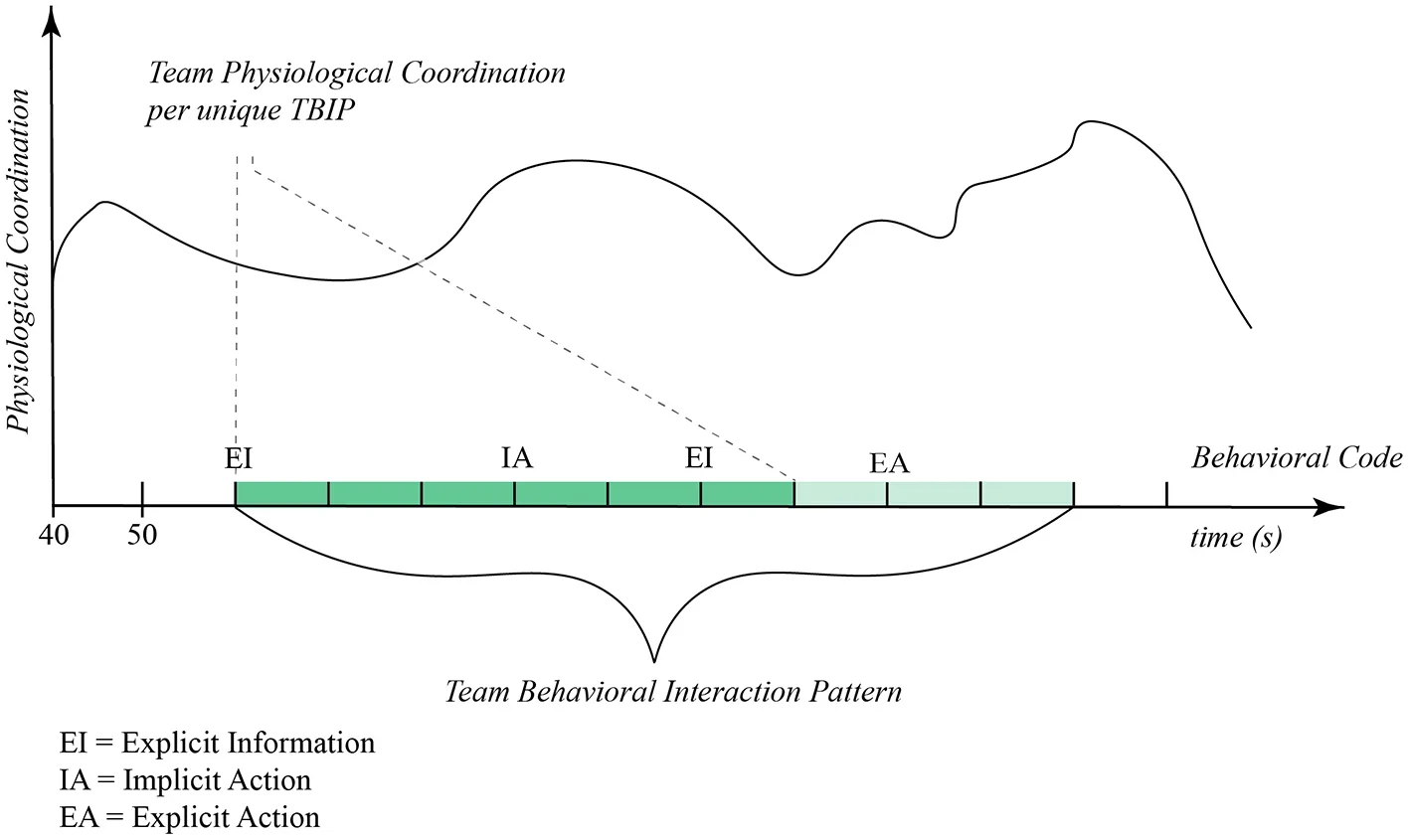
Visualization of an example of TBIP extraction and the calculation of team physiological coordination per unique pattern. Light green shading indicates the full duration of the identified TBIP, while dark green highlights the 60-s window used to calculate team physiological coordination for that pattern.

Subsequently, we calculated the mean physiological coordination for each type of TBIP within a given scenario by averaging across all occurrences of that pattern within that scenario.

#### 2.4.3 Team adaptation

Participants filled in a shortened questionnaire on team adaptive performance (Marques-Quinteiro et al., 2015), consisting of three team-based items, each rated on a 5-point Likert scale from 1 = *Totally Disagree* to 5 = *Totally Agree*. To facilitate interpretation, scores were linearly transformed to a 0–100 scale, where 0 represents the minimum possible score and 100 the maximum possible score. All reported descriptive statistics and analyses are based on these transformed scores. An example item was: “We adjust and deal with unpredictable situations by shifting focus and taking reasonable action.” Cronbach's alpha was equal to 0.8. To investigate whether data could be aggregated from the individual to the team level, we used the ‘multilevel' package in R to calculate *ICC*_(1)_ = 0.10, *ICC*_(2)_ = 0.41, and *rWG*_(*j*)_ = 0.94. The *ICC*_(1)_ indicated that a meaningful proportion of the variance was attributable to team membership, whereas the *ICC*_(2)_ indicated modest reliability of the aggregated team means. Still, the *rWG*_(*j*)_ value demonstrated strong within-team agreement, supporting the aggregation of individual adaptation scores to the team level.

#### 2.4.4 Team performance

Consistent with approaches to performance assessment in simulation-based team training, we conceptualized team performance as the observable behaviors through which teams manage the clinical task, rather than solely as the final clinical outcomes, which reflect team effectiveness (Rosen et al., 2010; Shapiro et al., 2008). Trainers assessed the teams' performance at the end of each scenario using a shortened version of the previously validated Communication and Teamwork Skills Assessment scale (Frankel et al., 2007). We removed two items (“briefing” and “debriefing”) that were not relevant to the current study, resulting in a scale with four categories and 19 items in total. Each item related to a performance indicator that was to be rated on a three-point scale: 1 = *Observed and Good*, 2 = *Observed and Marginal*, and 3 = *Expected but not Observed*. Scores were reverse-coded so that higher values reflected better performance. Again, to facilitate interpretation, scores were linearly transformed to a 0–100 scale, where 0 represents the minimum possible score and 100 the maximum possible score. All reported descriptive statistics and analyses are based on these transformed scores. An example item was: “Coordination: verbally identify who is in charge.” Cronbach's alpha was equal to 0.89.

### 2.5 Data analysis

All data analyses were preregistered in the OSF (https://osf.io/x9tph/). All analyses were performed in R (R Core Team, 2024) using RStudio. To address RQ1, we analyzed data at the scenario level. More specifically, we ran regression analyses using the built-in *stats* package to examine how the complexity of team interaction patterns (TBIPs) within a given scenario was related to team adaptation and team performance in that scenario. We included hospital as a categorical variable to account for potential differences in trainers and training practices across the two sites. To address RQ2, we analyzed data at the TBIP level. We used multilevel linear models (lmer, from the *lme4* package; Bates et al., 2015) to explore whether team physiological coordination during TBIPs was related to team adaptation or team performance, and whether these relationships depended on TBIP complexity. We used multiple measures of physiological coordination during TBIPs as dependent variables, as this allowed us to examine whether physiological coordination was associated with team outcomes and whether these associations varied with TBIP complexity. The specification of physiological coordination as the dependent variable reflected the multilevel structure of the data and was not intended to imply a causal or temporal direction between physiological coordination and team outcomes.

Since each team contributed multiple measurements, i.e., one per TBIP type per scenario, data were nested within teams. To account for this, we used mixed-effects models with a maximal random-effects structure (Barr et al., 2013), including random intercepts and slopes for each team. In the fixed effects, we entered either team adaptation or team performance, TBIP complexity, and their interaction to examine whether the association between team outcomes and physiological coordination varied across levels of TBIP complexity. If a model failed to converge, we removed the random slope, and if necessary, the random intercept for team. We also tested for a hospital-level random intercept, but hospital variance was near zero. Consequently, all final models included only random intercepts for scenarios to account for repeated measures. Standardized parameters were obtained by fitting the model on a standardized version of the dataset. 95% confidence intervals (CIs) and *p*-values were computed using a Wald t-distribution approximation.

## 3 Results

Two scenarios were excluded from the analysis as no TBIPs were detected. Across the remaining scenarios and teams, a total of 3,186 TBIPs were identified, comprising 773 unique patterns. Descriptive statistics, as shown in Table 1, indicated a minimum of eight and a maximum of 394 patterns per scenario, with a mean of 91.03 (*SD* = 102.18). After removing patterns shorter than 30 s, 496 patterns remained in the final dataset. Across scenarios, the number of unique TBIPs per scenario ranged from 1 to 81, with an average of 14.59 (*SD* = 20.72). The total count of TBIP instances per scenario ranged from 1 to 234 (*M* = 38.03, *SD* = 57.23). The identified TBIPs varied in length, hierarchical complexity, behavioral diversity (number of behavioral quadrants represented), and the number of transitions between behavioral quadrants. Specifically, TBIPs ranged from 2 to 13 behavioral elements (*M* = 4.42, *SD* = 2.03) and from complexity levels 1 to 7 (*M* = 2.83, *SD* = 1.4). Patterns contained between 1 and 4 behavioral quadrants (*M* = 2.33, *SD* = 0.81) and between 0 and 9 quadrant switches (*M* = 1.72, *SD* = 1.23). Individual patterns occurred between 1 and 9 times (*M* = 2.61, *SD* = 0.98). Additional descriptive statistics and correlations of the study variables at the TBIP and scenario levels are presented in Table 2.

**Table 1:** Descriptive statistics of average TBIP metrics per scenario (*N* = 35).

	Retrieved from the full sample (*N* = 3186)				Only TBIPs of 30 s or more (*N* = 1293)				TBIPs used for analyses: only the first occurrence of TBIPs of 30 sec or more (*N* = 496)			
	M	SD	Min	Max	M	SD	Min	Max	M	SD	Min	Max
Number of TBIPs	91.03	102.18	8	394	38.03	57.23	1	234	14.59	20.72	1	81
Number of unique TBIPs	22.09	25.62	2	101	14.59	20.72	1	81	14.59	20.72	1	81
Length of the TBIP	2.65	0.67	2	4.73	3.24	1.08	2	6.38	3.19	1.03	2	6.23
Complexity of the TBIP	1.54	0.50	1	2.95	2.02	0.77	1	3.95	1.99	0.74	1	3.85
Number of behavioral quadrants in each TBIP	1.63	0.33	1	2.42	1.91	0.51	1	2.8	1.89	0.49	1	2.79
Number of switches between behavioral quadrants	0.71	0.43	0	1.82	1.06	0.69	0	2.59	1.03	0.65	0	2.52

**Table 2:** Descriptive Statistics and Correlations for all Study Variables at Scenario Level (*N* = 35) and TBIP Level (*N* = 496).

Variable	*M*	*SD*	1	2	3	4	5	6	7
1. Team adaptation	67.34	7.53		0.14^**^	0.17^***^	−0.29^***^	0.11^*^	0.28^***^	−0.31^***^
2. Team performance	69.93	13.61	0.18		0.64^***^	0.04	0.13^**^	0.23^***^	0.18^***^
3. TBIP complexity	1.54	0.50	0.17	0.64^***^		−0.07	0.25^***^	0.23^***^	0.29^***^
4. Synchrony HR	0.21	0.02	−0.08	0.30	0.26		−0.04	−0.04	0.10^*^
5. Regularity HR	0.75	0.18	0.26	0.11	0.06	0.02		0.19^***^	0.19^***^
6. Synchrony EDA	0.17	0.01	0.27	0.11	0.12	−0.19	0.17		0.11^*^
7. Regularity EDA	0.40	0.16	0.12	0.26	0.34^*^	0.15	0.03	−0.06	

TBIP, Team behavioral interaction pattern. The lower triangle presents correlations at the scenario level, whereas the upper triangle presents correlations at the TBIP level. ^*^ indicates *p* < 0.05, ^**^ indicates *p* < 0.01, ^***^ indicates *p* < 0.001.

We ran two regression analyses to address RQ1, examining whether TBIP complexity related to team adaptation and team performance. The results are presented in Table 3. Regarding team adaptation, the first model predicting adaptation from TBIP complexity (β = 0.21, *p* = 0.22) was not significant, *F*_(1, 33)_ = 1.56, *p* = 0.22, *R*^2^ = 0.05. Adding hospital as a fixed-effect covariate to adjust for potential trainer or site differences in the second model did not improve model fit, *F*_(2, 32)_ = 0.76, *p* = 0.48, *R*^2^ = 0.05

**Table 3:** Standardized results of the regression analyses for team adaptation and team performance (*N* = 35).

	*Variable*	*B*	*SE*	*t*	95% CI	*p*
*Team Adaptation*						
Model 1	TBIP complexity	0.21	0.17	1.25	−0.13, 0.56	0.22
Model fit	*R* ^2^	Adjusted *R*^2^	*F*	*P*		
	0.05	0.02	1.56	0.22		
Model 2	TBIP complexity	0.23	0.25	0.89	−0.29, 0.74	0.38
	Hospital	−0.04	0.55	−0.08	−1.17, 1.09	0.94
Model fit	*R* ^2^	Adjusted *R*^2^	*F*	*P*		
	0.05	0	0.76	0.48		
*Team Performance*						
Model 1	TBIP complexity	0.63	0.14	4.64	0.35, 0.90	< 0.001
Model fit	*R* ^2^	Adjusted *R*^2^	*F*	*P*		
	0.40	0.38	21.57	< 0.001		
Model 2	TBIP complexity	0.003	0.13	0.02	−0.27, 0.28	0.98
	Hospital	1.86	0.29	6.38	1.27, 2.46	< 0.001
Model fit	*R* ^2^	Adjusted *R*^2^	*F*	*P*		
	0.73	0.72	44.12	< 0.001		

The first model predicting team performance was significant overall, *F*_(1, 33)_ = 21.57, *p* < 0.001, *R*^2^ = 0.40, but after adjusting for hospital in the second model [*F*_(2, 32)_ = 44.12, *p* < 0.001, *R*^2^ = 0.73], TBIP complexity was not significant (β = 0.003, *p* = 0.98). Hospital was strongly predictive of performance (β = 1.86 *p* < 0.001), consistent with performance differences between sites. Thus, even though at the zero-order level, higher mean TBIP complexity was positively associated with team performance, this association was no longer significant after controlling for the site at which data were collected. The disappearance of this relationship after controlling for site may reflect variation in trainer ratings across hospitals. However, because multiple factors differed between sites, including training structure and team composition, the specific source of this effect cannot be determined from the present data.

To address RQ2, which asked whether team physiological coordination during TBIPs relates to team adaptation or team performance, and whether these relationships depend on TBIP complexity, we first calculated zero-order correlations between these variables, which can be found in Table 1. Next, we ran four sets of models with physiological coordination as the dependent variable, namely heart rate synchrony, skin conductance synchrony, heart rate regularity, and skin conductance regularity. Within each set, one model examined team adaptation and one examined team performance as predictors; results are displayed in Tables 4 and 5, respectively.

**Table 4:** Standardized results of the multilevel analyses for team adaptation for models 1 and 2 (*N* = 496).

	Model 1					Model 2				
Fixed effect	β	*SE*	*t*	*p*	95% CI	β	*SE*	*t*	*p*	95% CI
*HR Synchrony*										
Intercept	−0.08	0.12	−0.68	0.50	−0.33, 0.16	−0.09	0.12	−0.70	0.49	−0.32, 0.15
Team adaptation	0.19	0.13	1.43	0.16	−0.07, 0.46	0.13	0.13	0.99	0.32	−0.13, 0.39
TBIP complexity	0.03	0.04	0.74	0.46	−0.05, 0.12	0.01	0.04	0.24	0.81	−0.08, 0.10
Team adaptation × TBIP complexity						–**0.16**	**0.04**	–**3.93**	**< 0.001**	−0**.24**, −0**.08**
Random effect	Variance		SD			Variance		SD		
Intercept	0.34		0.58			0.33		0.57		
Model fit	Conditional *R*^2^		Marginal *R*^2^			Conditional *R*^2^		Marginal *R*^2^		
	0.35		0.04			0.35		0.04		
*EDA Synchrony*										
Intercept	−0.06	0.13	−0.46	0.65	−0.30, 0.19	−0.06	0.12	−0.47	0.64	−0.30, 0.18
Team adaptation	−0.10	0.14	−0.75	0.45	−0.37, 0.16	−0.17	0.13	−1.24	0.21	−0.43, 0.10
TBIP complexity	0.04	0.04	0.95	0.34	−0.04, 0.12	0.02	0.04	0.42	0.67	−0.07, 0.10
Team adaptation × TBIP complexity						–**0.16**	**0.04**	–**4.21**	**<** **0.001**	**−0.24** **−0.09**
Random effect	Variance		SD			Variance		SD		
Intercept	0.36		0.6			0.34		0.59		
Model fit	Conditional *R*^2^		Marginal *R*^2^			Conditional *R*^2^		Marginal *R*^2^		
	0.36		0.01			0.4		0.07		
*HR Regularity*										
Intercept	−0.53	0.25	−2.15	0.03	−1.01, −0.05	−0.53	0.25	−2.14	0.03	−1.02, −0.04
Team adaptation	0.17	0.27	0.64	0.53	−0.36, 0.70	0.19	0.27	0.71	0.48	−0.34, 0.73
TBIP complexity	0.03	0.03	0.79	0.43	−0.04, 0.09	0.03	0.03	0.97	0.33	−0.03, 0.09
Team adaptation × TBIP complexity						0.04	0.03	1.43	0.15	−0.02, 0.10
Random effect	Variance		SD			Variance		SD		
Intercept	1.85		1.36			1.88		1.37		
Model fit	Conditional *R*^2^		Marginal *R*^2^			Conditional *R*^2^		Marginal *R*^2^		
	0.84		0.01			0.85		0.02	
*EDA Regularity*										
Intercept	−0.17	0.16	−1.10	0.27	−0.49, 0.14	−0.17	0.16	−1.10	0.27	−0.49, 0.14
Team adaptation	0.29	0.17	1.68	0.09	−0.05, 0.63	0.29	0.17	1.69	0.09	−0.05, 0.63
TBIP complexity	0.05	0.03	1.67	0.10	−0.01, 0.12	0.06	0.03	1.68	0.09	−0.01, 0.12
Team adaptation × TBIP complexity						0.01	0.03	0.19	0.85	−0.05, 0.07
Random effect	Variance		SD			Variance		SD		
Intercept	0.7		0.84			0.7		0.84		
Model fit	Conditional *R*^2^		Marginal *R*^2^			Conditional *R*^2^		Marginal *R*^2^		
	0.68		0.08			0.68		0.08		

Bold numbers indicate significant relationships.

**Table 5:** Standardized results of the multilevel analyses for team performance for models 1 and 2 (*N* = 496).

	Model 1					Model 2				
Fixed effect	β	*SE*	*t*	*p*	95% CI	β	*SE*	*t*	*p*	95% CI
*HR Synchrony*										
Intercept	−0.05	0.15	−0.32	0.75	−0.33, 0.24	−0.03	0.15	−0.20	0.85	−0.32, 0.26
Team performance	0.09	0.11	0.82	0.41	−0.13, 0.30	0.06	0.12	0.49	0.62	−0.18, 0.29
TBIP complexity	0.03	0.05	0.66	0.51	−0.06, 0.12	0.05	0.05	0.88	0.38	−0.06, 0.15
Team performance × TBIP complexity						−0.04	0.07	−0.64	0.52	−0.18, 0.09
Random effect	Variance		SD			Variance		SD		
Intercept	0.35		0.59			0.36		0.60		
Model fit	Conditional *R*^2^		Marginal *R*^2^			Conditional *R*^2^		Marginal *R*^2^		
	0.34		0.01			0.34		0.009		
*EDA Synchrony*										
Intercept	0.07	0.14	0.50	0.26	−0.21, 0.36	0.06	0.15	0.41	0.68	−0.23, 0.35
Team performance	0.16	0.11	1.51	0.13	−0.05, 0.38	0.19	0.12	1.58	0.12	−0.05, 0.42
TBIP complexity	0.03	0.04	0.75	0.45	−0.05, 0.12	0.02	0.05	0.42	0.67	−0.08, 0.12
Team performance × TBIP complexity						0.03	0.06	0.49	0.63	−0.09, 0.16
Random effect	Variance		SD			Variance		SD		
Intercept	0.35		0.59			0.35		0.59		
Model fit	Conditional *R*^2^		Marginal *R*^2^			Conditional *R*^2^		Marginal *R*^2^		
	0.37		0.03			0.37		0.03		
*HR Regularity*										
Intercept	−0.27	0.30	−0.90	0.37	−0.85, 0.32	−0.25	0.30	−0.85	0.40	−0.84, 0.33
Team performance	0.34	0.21	1.58	0.12	−0.08, 0.76	0.31	0.22	1.43	0.15	−0.12, 0.73
TBIP complexity	0.02	0.03	0.74	0.46	−0.04, 0.09	0.04	0.04	1.02	0.31	−0.03, 0.11
Team performance × TBIP complexity						−0.04	0.05	−0.76	0.45	−0.13, 0.06
Random effect	Variance		SD			Variance		SD		
Intercept	1.73		1.30			1.70		1.30		
Model fit	Conditional *R*^2^		Marginal *R*^2^			Conditional *R*^2^		Marginal *R*^2^		
	0.84		0.05			0.84		0.05	
*EDA Regularity*										
Intercept	−0.14	0.20	−0.71	0.48	−0.54, 0.25	−0.20	0.20	−0.99	0.32	−0.59, 0.19
Team Performance	0.10	0.15	0.67	0.50	−0.19, 0.38	0.20	0.15	1.33	0.18	−0.09, 0.49
TBIP Complexity	0.05	0.03	1.63	0.10	−0.01, 0.12	0.00	0.04	0.09	0.93	−0.07, 0.08
Team Performance × TBIP Complexity						**0.13**	**0.05**	**2.67**	**0.008**	**0.04, 0.23**
Random effect	Variance		SD			Variance		SD		
Intercept	0.76		0.87			0.74		0.86		
Model fit	Conditional *R*^2^		Marginal *R*^2^			Conditional *R*^2^		Marginal *R*^2^		
	0.67		0.01			0.67		0.03		

Bold numbers indicate significant relationships.

First, we ran two sets of linear mixed-effects models to examine how physiological coordination metrics were predicted by team adaptation or team performance, with TBIP complexity included as an additional fixed effect. In each model, TBIP complexity and either team adaptation or performance were entered as main effects. No significant main effects of TBIP complexity, team adaptation, or team performance on any of the physiological coordination metrics were found.

We then added an interaction term (either between team adaptation and TBIP complexity or between performance and TBIP complexity) to determine whether the relationships between physiological coordination and team adaptation or team performance depended on TBIP complexity. Two interaction effects between team adaptation and TBIP complexity were statistically significant, and one interaction effect between TBIP complexity and team performance.

The model predicting HR synchrony from team adaptation, TBIP complexity, and their interaction showed a conditional *R*^2^ of 0.35 and a marginal *R*^2^ of 0.40, indicating that the overall model explained 35% and the fixed effects explained 4% of the variance in HR synchrony. We found no main effect but the interaction between team adaptation and TBIP complexity was significant and negative (β = −0.16, *p* < 0.001).

Similarly, the model predicting EDA synchrony from team adaptation, TBIP complexity, and their interaction showed a conditional *R*^2^ of 0.40 and a marginal *R*^2^ of 0.07. The overall model thus explained 40% and the fixed effects explained 7% of the variance in EDA synchrony. Here too, we found no main effect, but the interaction between team adaptation and TBIP complexity was significant and negative (β = −0.16, *p* < 0.001).

The model predicting EDA regularity from team performance, TBIP complexity, and their interaction showed a conditional *R*^2^ of 0.67, indicating that the model explained 67% of the variance in the outcome while the fixed effects alone accounted for 3% of the variance (marginal *R*^2^ = 0.03). We found no main effect, but the interaction between team performance and TBIP complexity was statistically significant and positive (β = 0.13, *p* = 0.008).

To further probe the significant interactions, we conducted Johnson–Neyman analyses to identify the regions of TBIP complexity at which the effects of team adaptation or team performance on physiological coordination were statistically significant. For HR synchrony, the relationship with team adaptation was significant when TBIP complexity was more than 0.86 SD below the mean. For EDA synchrony, the relationship with team adaptation became significant when TBIP complexity was 0.72 SD above the mean. For EDA regularity the relationship with team performance became significant when TBIP complexity was 1.13 SD above the mean. These effects are visualized in Figure 4.

**Figure 4:**
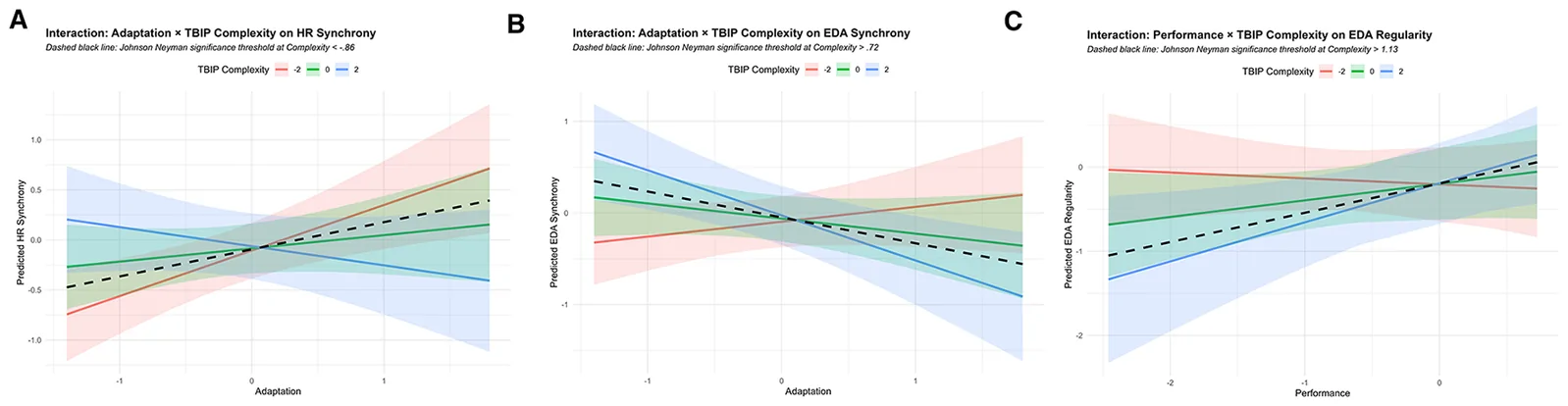
Plots illustrating the significant conditional effects of team adaptation and team performance on physiological coordination as a function of TBIP complexity. Predicted values of are plotted at low (−2 SD), mean (0), and high (+2 SD) levels of TBIP complexity. In (**A** and **B**), team adaptation significantly predicted HR synchrony and EDA synchrony respectively. **(C)**, Team performance significantly predicted EDA regularity. Solid lines represent estimated slopes; shaded bands indicate 95% confidence intervals. Vertical dashed lines denote regions of statistical significance (*p* < 0.05).

In sum, the relationship between physiological synchrony and team adaptation varied depending on TBIP complexity. Specifically, greater HR synchrony was related to greater team adaptation when TBIP complexity was low, whereas lower EDA synchrony was related to greater team adaptation only when TBIP complexity was high. Thus, HR and EDA synchrony were both related to team adaptation, but in different ways depending on the complexity of TBIPs. EDA regularity was related to team performance, but only at higher levels of TBIP complexity, where greater regularity in EDA signals was associated with better team performance.

## 4 Discussion

There is an increasing need and acceptance for providing teams, especially action teams working in high-stakes environments, with timely and reliable feedback to support their team performance (Allemang-Trivalle, 2023; Wespi et al., 2023). Advances in wearable (bio) sensors now allow for continuous monitoring of physiological signals creating new opportunities for real-time team assessment and support (Kadian et al., 2023; van Eijndhoven et al., 2023). Building on these developments, this research aimed to contribute to understanding the potential role of continuous measurement of team physiological coordination in assessing team functioning in dynamic crises contexts.

We evaluated whether team adaptation and team performance of medical teams in crisis simulations were related to the complexity of their behavioral interaction patterns and to what extent the complexity of these patterns affected the relationship between team outcomes and physiological coordination. To investigate this, we conducted a quasi-experimental study in which teams of medical professionals participated in simulation-based medical team training designed to prepare them for high-stakes medical emergencies. Across scenarios, we found no evidence of a significant relationship between TBIP complexity and team adaptation or team performance, but at at a more fine-grained level, TBIP complexity proved important for linking physiological coordination with team adaptation and team performance. Specifically, the positive relation between heart rate synchrony and team adaptation was only significant for low-complexity TBIPs, while the relationship between skin conductance synchrony and team adaptation was significant for high-complexity TBIPs. The relation between regularity in skin conductance and team performance was only significant for highly complex TBIPs.

As such, the current study provides important insights regarding the contribution of TBIPs to team adaptation and team performance, and informs future research on the potential use of wearable technology for assessing team functioning in high-stakes scenarios.

### 4.1 Theoretical implications

First, our findings indicated that there was no significant relationship between TBIP complexity and team adaptation or performance. This contrasts with earlier work in which more effective crews displayed stable levels of TBIP complexity (Zijlstra et al., 2012) and more homogeneous TBIPs (Kanki et al., 1989). However, those studies examined highly trainable routine simulator tasks in an aviation context, where adaptation demands were limited in contrast to the high-pressure medical simulations used in the current study. Medical teams develop and rehearse specific TBIPs to respond effectively to familiar disruptions, but also face disruptions that are novel, obscure, highly unexpected or not yet recognized. As such, medical teams often follow standardized protocols but also improvise when unexpected events occur, thereby engaging in so-called ambidextrous coordination, drawing on both flexibility and stability in team interaction (Grote et al., 2018). Prior research showed that teams relied on simpler, well-practiced behavioral interaction patterns in routine situations (Stachowski et al., 2009), but engaged in more complex TBIPs in the context of more novel or unexpected scenarios (David et al., 2024). In the context of our study, it is plausible that medical teams engaged in ambidextrous coordination with high- and low-complexity TBIPs, where neither was universally beneficial for team adaptation or performance. Instead, varying degrees of TBIP complexity may be necessary to balance stability and flexibility for effective adaptive performance in high-stakes situations (Grote et al., 2018).

This interpretation aligns with the Law of Requisite Variety (Ashby, 1957), which proposes that adaptive systems require sufficient internal variety to match the complexity of environmental demands. That is, teams may adapt the complexity of TBIPs in response to contextual requirements, relying on simpler patterns in stable and predictable situations while increasing behavioral variety when confronted with uncertainty or unexpected events. In fact, Grimm et al. (2023) demonstrated that increasing environmental demands were associated with greater reorganization at the team level. Related work has shown that complexity matching can emerge in various coordination contexts, including dyadic conversations (Abney et al., 2014), movement coordination (Marmelat and Delignières, 2012), side-by-side walking (Almurad et al., 2017), and collective activities such as rowing (Den Hartigh et al., 2018). As such, team theory should consider that both simple and complex TBIPs may be important for team adaptation and performance, and that their relative value may depend on the nature of the crisis.

Second, our findings showed that physiological coordination was associated with adaptive team functioning, but that these associations were not uniform and depended on TBIP complexity. Complex and simple TBIPs may reflect different cognitive, affective, or behavioral demands that require distinct forms of alignment across system levels, which may be partially observed in physiological coordination. More specifically, high-complexity patterns likely involve greater cognitive demands, as they require teams to execute longer sequences of interdependent actions in a specific order. In these cases, greater physiological regularity may reflect heightened cognitive workload (Hałgas, 2026) or sustained focus (Dindar et al., 2020), which could support effective execution and thus benefit performance. This could explain the positive relationship we found between physiological regularity and team performance for complex TBIPs.

For high-complexity TBIPs, lower skin conductance synchrony was associated with higher team adaptation. This result may reflect the role of creative processes in enabling teams to adjust flexibly under demanding conditions. Specifically, high-complexity TBIPs often require teams to move beyond well-established routines. Such shifts can be supported by a process of “desynchronization,” a temporary disruption of stable coordination that increases uncertainty and enables reorganization (Laroche et al., 2024). Moreover, lower neural synchrony has been linked to higher creativity in group tasks (Liang et al., 2022). Taken together, these findings suggest that more complex TBIPs may demand creative reorganization, and that lower physiological synchrony may reflect this process and support team adaptation.

In contrast, high heart rate synchrony was related to better team adaptation, but only for low-complexity TBIPs. Less complex TBIPs may reflect routinized interactions that rely on shared mental models. When teams repeatedly act on such models, their coordination becomes increasingly automated and cognitively efficient (Cannon-Bowers et al., 1993; Salas et al., 1995). Thus, in the context of our study, higher physiological synchrony for low-complexity TBIPs may reflect lower cognitive load and enhanced coordination efficiency, as team members operate based on the same expectations with minimal need for explicit communication (Henning et al., 2009). Thus, it is possible that in routine conditions, physiological synchrony may reflect automatic interactions based on shared mental models. However, the present study did not directly assess implicit coordination or communication frequency, and this interpretation therefore remains speculative. Future research could therefore examine how the behavioral content of TBIPs, such as the prevalence of implicit coordination, speaking up behaviors, or information exchange, relates to both TBIP complexity and physiological coordination.

The different associations observed for heart rate and skin conductance synchrony for low- and high-complexity patterns might be linked to the sensitivity of these signals to different team processes and states. Heart rate has been linked to attentional processes, mental effort, and task engagement (Fairclough, 2009; Mehler et al., 2009; Roscoe, 1992), whereas electrodermal activity primarily reflects sympathetic arousal and stress-related activation (Critchley, 2002; Healey and Picard, 2005). In the present study, heart rate synchrony was associated with better adaptation during low-complexity TBIPs perhaps reflecting routinized interactions supported by shared mental models, allowing team members to coordinate efficiently with limited explicit communication (Cannon-Bowers et al., 1993; Henning et al., 2009; Salas et al., 1995). In contrast, lower skin conductance synchrony was associated with better adaptation during high-complexity TBIPs, when teams may encounter novel demands that require them to move beyond established routines. Under such conditions, lower skin conductance synchrony may reflect a process of reorganization and exploration that supports team adaptation (Laroche et al., 2024). Together, these findings suggest that physiological coordination is not inherently beneficial or detrimental, but depends on the behavioral context in which it occurs.

Taken together, our research integrates and contributes to two streams of the team literature, one related to TBIPs and the other to physiological coordination, advancing understanding of how physiological coordination and behavioral complexity interact to support team adaptation and team performance. Specifically, our findings suggest that associations between physiological coordination and team adaptation or team performance depend on the behavioral interaction context in which physiological coordination occurs. As such, to support team performance under varying forms of crisis, our results highlight the need for integrated approaches and theoretical models that combine situational context with physiological and behavioral data.

### 4.2 Practical implications and future directions

Our findings underscore the importance of combining behavioral data with team physiological coordination to better understand team adaptation and team performance. Specifically, depending on the type of crisis a team faces, whether it is novel or routine, the complexity of behavioral patterns may shift. Practically, this suggests that both standardized responses, which may rely on low-complexity interaction patterns, and novel responses, which may involve high-complexity interaction patterns, are essential for supporting team adaptation and performance. Future research could examine how effective teams balance adherence to, and deviation from, standard procedures, and how they switch between simple and complex behavioral patterns in response to changing contexts. Relatedly, future research could examine how different structural characteristics of TBIPs, such as hierarchical organization, the number of constituent behavioral elements, and pattern duration jointly contribute to the complexity of naturally occurring team interaction patterns. Furthermore, research could examine whether team adaptation depends on the match between interaction complexity and environmental demands. Because task demands may differ across teams and change as a scenario unfolds, future work should treat them as a dynamic team-task property rather than a fixed scenario characteristic. This would require repeated assessment of experienced task demands alongside changes in TBIP complexity over time. Research could also investigate factors that may facilitate this process, such as leadership, trust, or shared mental models. Another direction concerns the role of team composition as team size, expertise, and prior experience may influence how teams respond to changing task demands and organize their interactions. Future research could investigate how team composition variables shape team physiological coordination dynamics and team adaptation in healthcare teams. Future research could also extend the assessment of team performance by complementing established behavioral measures with standardized clinical outcome measures, where these can be meaningfully defined and compared across scenarios.

Our results also show that physiological coordination may reflect team outcomes depending on the complexity of the TBIPs. As such, researchers might investigate whether parallel behavioral data streams (Allspaw et al., 2024) can support real-time pattern detection, although prospective validation and further development would be required before such approaches could be used for real-time team assessment. Another promising avenue is to examine whether teams that benefit from low- or high-complexity TBIPs also consistently show corresponding levels of physiological synchrony or regularity. It would also be valuable to investigate how teams transition between high- and low-complexity TBIPs, and whether such transitions are driven by situational demands and whether they are reflected in physiological coordination. Moreover, future research could examine the extent to which changes in behavioral complexity correspond to changes in task demands over time. From a requisite complexity perspective (Ashby, 1957), both simple and complex interaction patterns may be advantageous for team adaptation and performance, depending on the situation. As such, the extent to which teams adjust their behavioral complexity to match changing task demands, and how this alignment influences team adaptation and performance, represents a promising direction for future research. Such investigations would require continuous assessment of task complexity alongside time-varying analyses of behavioral complexity throughout team interaction.

Importantly, our findings suggest that skin conductance and heart rate, as well as measures of synchrony and regularity, capture different aspects of physiological coordination and may therefore provide complementary information about team functioning. Future research should examine whether combining these physiological measures provides a more comprehensive assessment of how teams function and adapt.

It is important to note, however, that the observed associations were relatively small, with the models accounting for approximately 3–7% of the variance. The present findings therefore provide evidence of contemporaneous associations rather than predictive validity. If future research aims to use physiological measures for real-time team monitoring, prospective and out-of-sample validation will be required. Such measures may ultimately prove most informative when combined with behavioral and contextual indicators, including leadership behavior, task complexity, or team experience. Future research may also benefit from examining the factors underlying variability in the number and diversity of detected TBIPs across teams and scenarios.

Due to the fact that women are often more strongly represented in the clinical specialties involved nursing, obstetrics, and gynecology (Statistics Netherlands, 2024) the sample consisted predominantly of female healthcare professionals. Previous research has suggested potential associations between gender and physiological coordination, yet findings remain mixed and have largely emerged from dyadic and emotionally focused paradigms rather than task-oriented team settings (e.g., Fujiwara et al., 2019). Therefore, it remains speculative whether gender composition influenced physiological coordination in the current study. Future research could investigate whether team gender composition influences team physiological coordination and its relationship with team adaptation and performance.

While the current state of the field does not yet allow for direct application of physiological signal monitoring for team support, the present findings identify directions for future research to evaluate whether physiological measures can ultimately contribute to real-time assessment in high-stakes settings.

### 4.3 Limitations

Despite its strengths, the current research has some limitations that warrant mention. First, we removed a portion of patterns based on duration thresholds, which may have influenced the representativeness of the remaining data, especially since shorter TBIPs are more likely to be less complex.

Second, differences in coding granularity may have influenced how TBIPs were detected in our study vs. previous work. For example, our coding scheme only allowed for coding of the occurrence of closed-loop communication (i.e., the event of closed loop communication being performed), while other studies might have coded it as a three-step behavior (i.e., message initiation, acknowledgment, and confirmation). Such differences in coding detail and granularity may have influenced both the number of patterns identified and their measured complexity, potentially limiting comparability across studies.

A third limitation concerns the preprocessing of the PPG data. Because the raw signal contained a non-trivial amount of noise, heart rate was estimated using a relatively long window of 120 s. Although this was necessary to obtain reliable estimates, it likely resulted in signal smoothing. Given the sliding windows used both for HR estimation from PPG and for the calculation of coordination, our operationalization of HR-based coordination primarily captures more slowly varying changes in HR. Moreover, some HR values contributing to a given coordination estimate fall outside the temporal boundaries of the corresponding TBIP. Consequently, HR-based coordination estimates were not fully restricted to the temporal boundaries of the corresponding TBIP, which may have reduced their sensitivity to TBIP-specific physiological dynamics.

Fourth, the limited sample size did not allow for more complex moderation analyses that could include other factors influencing the pattern complexity displayed by teams, such as task complexity, team experience, or team familiarity. The modest ICC(2) for team adaptation also indicates caution in interpreting the aggregated team-level adaptation scores. Future research could explore the role of additional variables shaping the relationship between team adaptation and team behavioral complexity.

Fifth, although healthcare simulations are designed to resemble clinical practice as closely as possible, they might not always reproduce the complexity, uncertainty, and consequences of actual patient care. Consequently, the relationships observed between TBIP complexity, physiological coordination, and team outcomes may differ in real-world clinical settings where stakes, workload, and contextual demands are higher. In addition, participants were aware that they were being video-recorded and monitored. Such awareness may introduce observer effects, whereby individuals modify their behavior because they know they are being monitored (Becker and Marique, 2014). Similarly, being observed may influence physiological responses, potentially affecting measures of physiological coordination. As such, there is a need for less obtrusive measurement in real-world scenarios to establish the generalizability of the present findings.

Lastly, the MdRQA analysis had to be performed using a 60 s window, as shorter windows did not provide enough data points for reliable detection on recurrence. Even though the coherence analyses did not require a fixed 60 s analysis window, we retained the same 60 s window used for the MdRQA analyses to ensure comparability between methods. It is therefore possible that, for shorter window lengths, the coherence results would show a clearer relationship with team adaptation or team performance. Future work could explore how different temporal resolutions affect the sensitivity of physiological coordination measures.

## 5 Conclusions

We examined how the complexity of a team's behavioral interaction patterns (TBIPs) relates to team adaptation and team performance, and whether the relationship between team outcomes (i.e., team adaptation and team performance) and physiological coordination (i.e., measures of synchrony and regularity) varies with TBIP complexity. Although TBIP complexity alone was not significantly associated with team outcomes, our results showed that the relationship between physiological coordination and team outcomes differed across levels of TBIP complexity. These findings suggest that physiological coordination may represent a context-sensitive correlate of team adaptation and team performance, although further validation is still needed. Importantly, our results demonstrated that TBIP complexity itself was not significantly associated with team adaptation or team performance, nor did we find a consistent relation between physiological coordination and team outcomes for all levels of behavioral complexity. Rather, our findings are consistent with the possibility that teams vary their use of TBIPs according to task demands and that physiological coordination may differ across these behavioral contexts. As such, rather than serving as a universal indicator of team adaptation or team performance, the relationships between physiological coordination and team adaptation and performance should be interpreted in relation to the behavioral context in which they occur. The present study therefore contributes to the growing literature on team physiological coordination by showing that associations between physiological coordination and team adaptation and performance vary across levels of behavioral complexity. Taken together, this work reinforces the need to develop dynamic models that capture how teams adapt over time, based on a combination of behavioral and physiological metrics. The presented results may inform future research on the development and validation of real-time team assessment systems, particularly those that aim to support medical teams in training or high-stakes operational environments. Future research should continue to explore how behavioral and physiological data streams can be integrated in real time to support teams facing evolving challenges, and should prospectively validate whether such integrated measures can reliably support real-time team assessment and feedback.

## Data Availability

The datasets presented in this article are not readily available due to privacy considerations. Requests to access anonymized data should be directed to the corresponding author.
